# Supplementary material for: Synthesized Bis-Triphenyl Phosphonium-Based Nano Vesicles Have Potent and Selective Antibacterial Effects on Several Clinically Relevant Superbugs
Source: Nanomaterials (Basel). 2024 Aug 15;14(16):1351. doi: 10.3390/nano14161351 (PMC11357385; doi:10.3390/nano14161351)
Supplement: Supplementary file 1 [file nanomaterials-14-01351-s001.zip › nanomaterials-3121322-supplementary.pdf]

Supplementary Materials

Synthesized *Bis*-Triphenyl Phosphonium-Based Nano Vesicles Have Potent and Selective Antibacterial Effects on Several Clinically Relevant Superbugs

Silvana Alfei [1,*], Guendalina Zuccari [1,2,*], Francesca Bacchetti [1], Carola Torazza [1], Marco Milanese [1,3], Carlo Siciliano [4], Constantinos M. Athanassopoulos [5], Gabriella Piatti [6] and Anna Maria Schito [6]

[1] Department of Pharmacy, University of Genoa, Viale Cembrano, 16148 Genoa, Italy; francesca.bacchetti@edu.unige.it (F.B.); carola.torazza@unige.it (C.T.); marco.milanese@unige.it (M.M.)

[2] Laboratory of Experimental Therapies in Oncology, IRCCS Istituto Giannina Gaslini, Via G. Gaslini 5, 16147, Genoa, Italy

[3] IRCCS Ospedale Policlinico San Martino, Genova, Italia

[4] Department of Pharmacy, Health and Nutritional Sciences, University of Calabria, 87036, Arcavacata of Rende, Italy; carlo.siciliano@unical.it

[5] Department of Chemistry, University of Patras, University Campus Rio Achaias, 26504 Greece; kath@chemistry.upatras.gr (C.M.A.)

[6] Department of Surgical Sciences and Integrated Diagnostics (DISC), University of Genoa, Viale Benedetto XV, 6, I-16132 Genova, Italy; amschito@unige.it (A.M.S); gabriella.piatti@unige.it (G.P.)

*Correspondence: alfei@difar.unige.it; Tel.: +39 010 355 2296 (S.A.); guendalina.zuccari@unige.it (G.Z.)

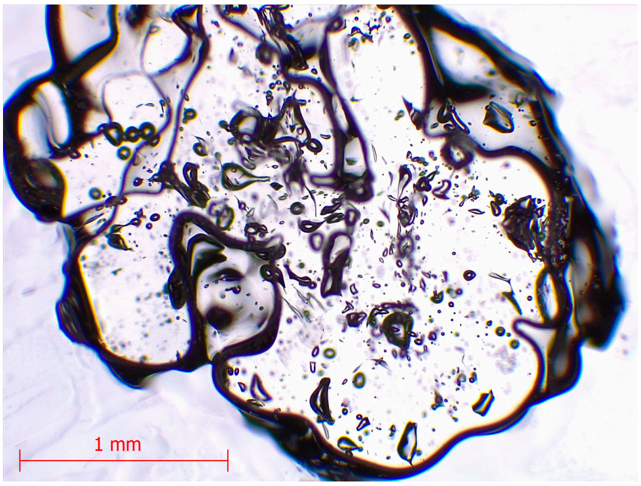

Figure S1. Optical micrograph of solid BPPB obtained using a 4 × objective.

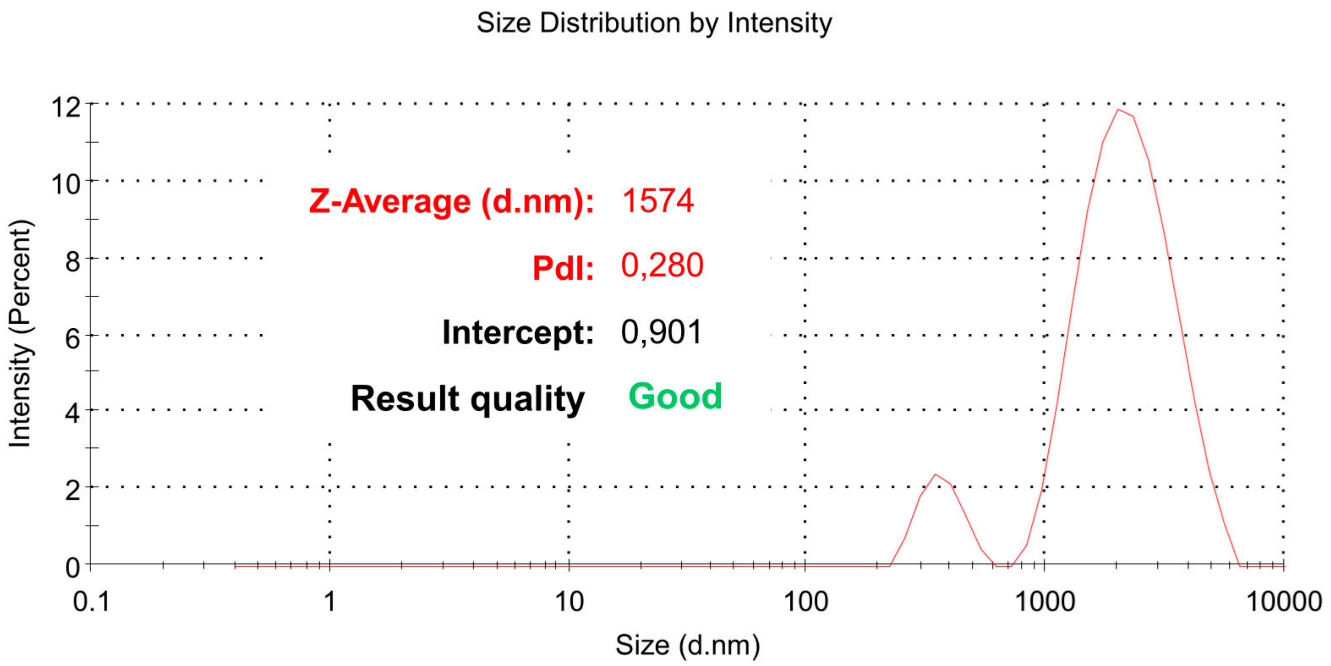

Figure S2. Size distribution of BPPB vesicles and large aggregates self-formed in a 10 mM solution.

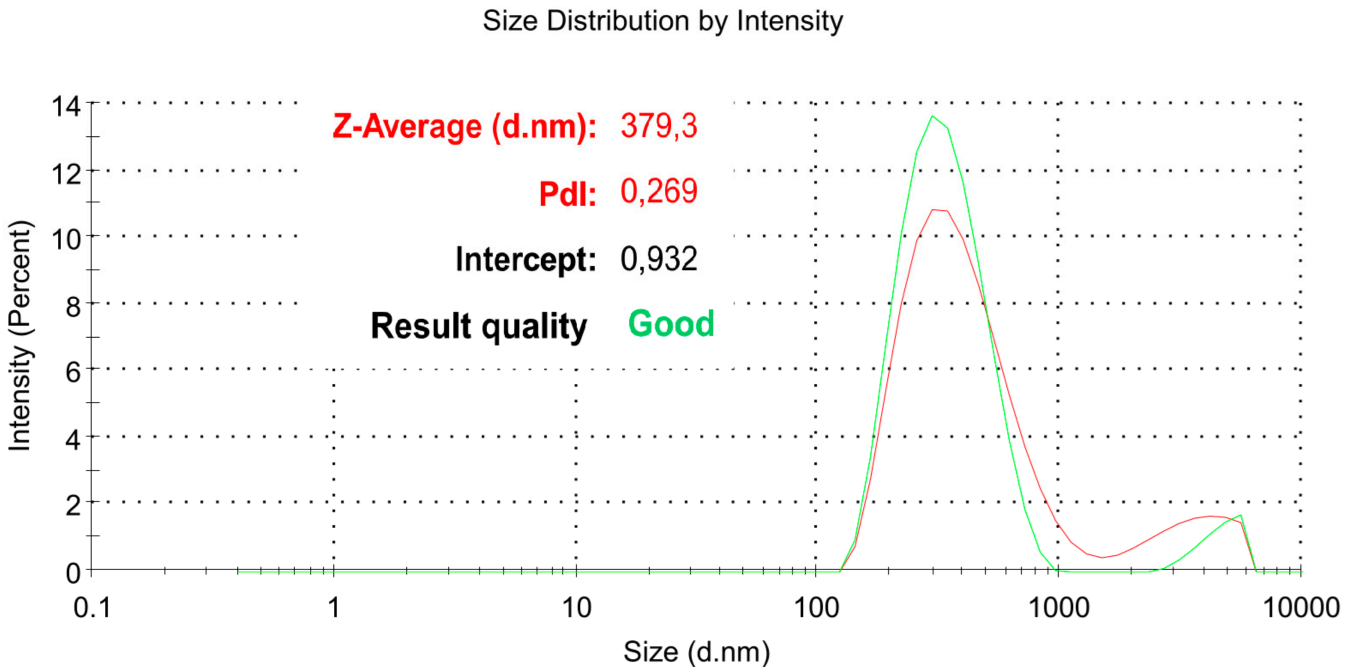

Figure S3. Size distribution of BPPB vesicles and aggregates self-formed in a 5 mM solution.

Results

			Size (d.nm...	% Intensity:	St Dev (d.n...
Z-Average (d.nm):	41,29	Peak 1:	77,92	77,9	32,86
PdI:	0,584	Peak 2:	1,605	11,3	0,1868
Intercept:	0,795	Peak 3:	12,86	5,8	2,470

Result quality Refer to quality report

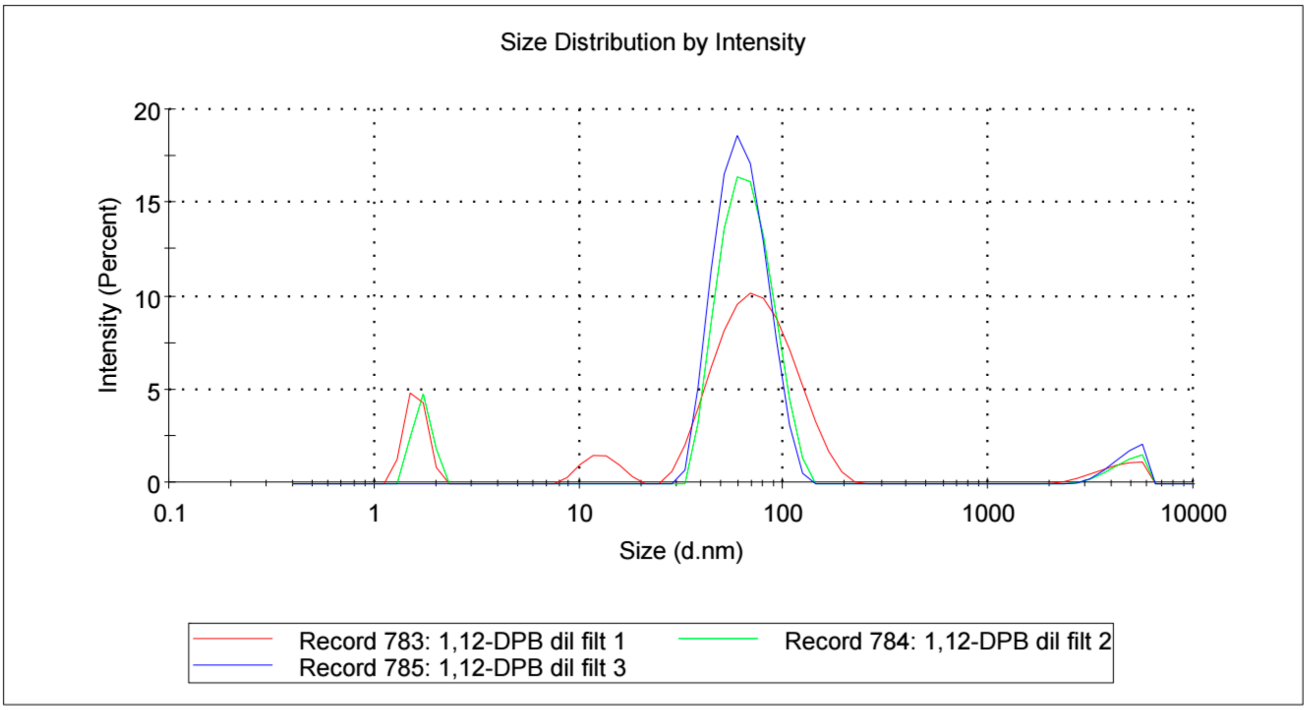

Figure S4. Size distributions of BPPB vesicles and aggregates self-formed in a 5 mM solution.

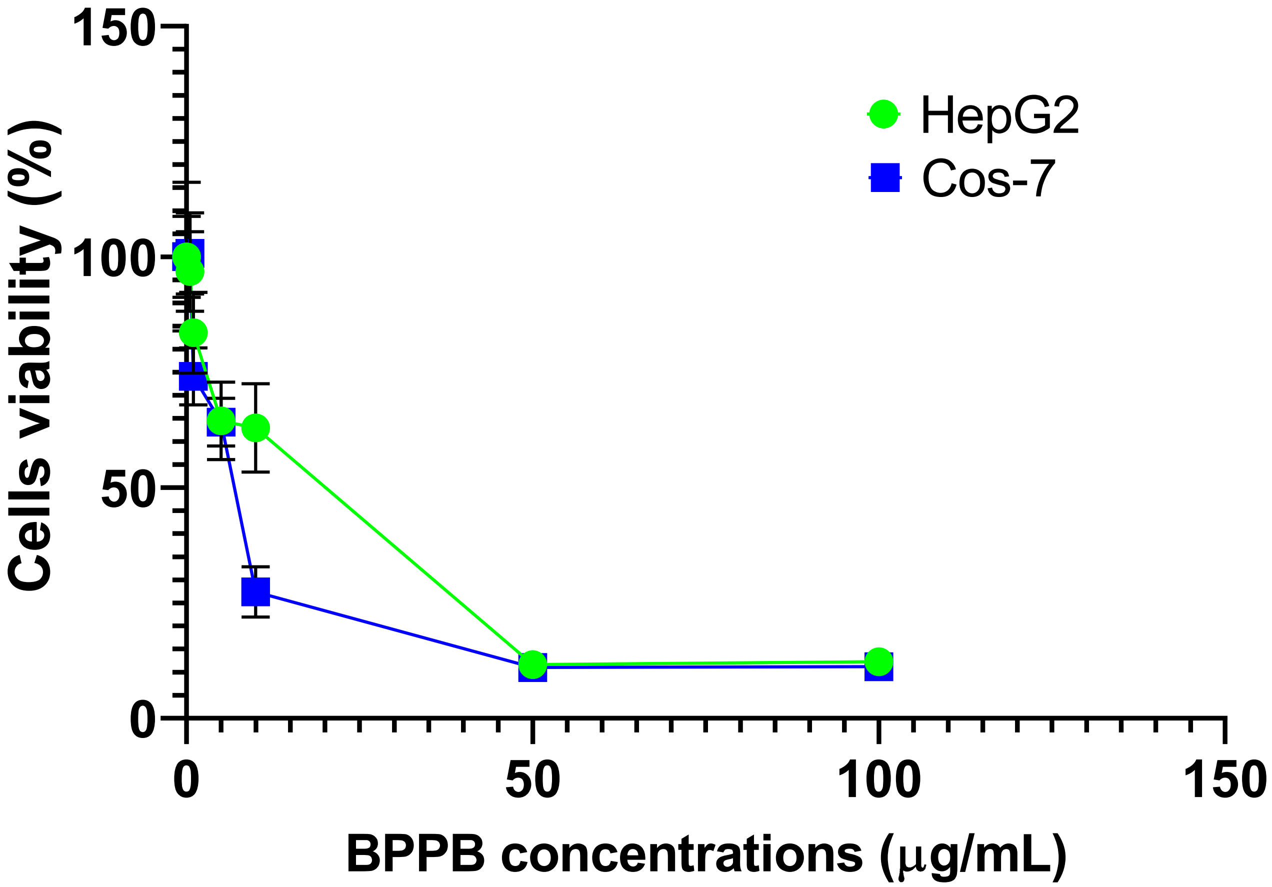

Figure S5. Cell viability (%) of Cos-7 (blue trace) and HepG2 (green trace) cells vs increasing BPPB concentrations (0.5-100 µg/mL) after 24 hours of exposure. Concentration = 0.0 µg/mL corresponded to the control.

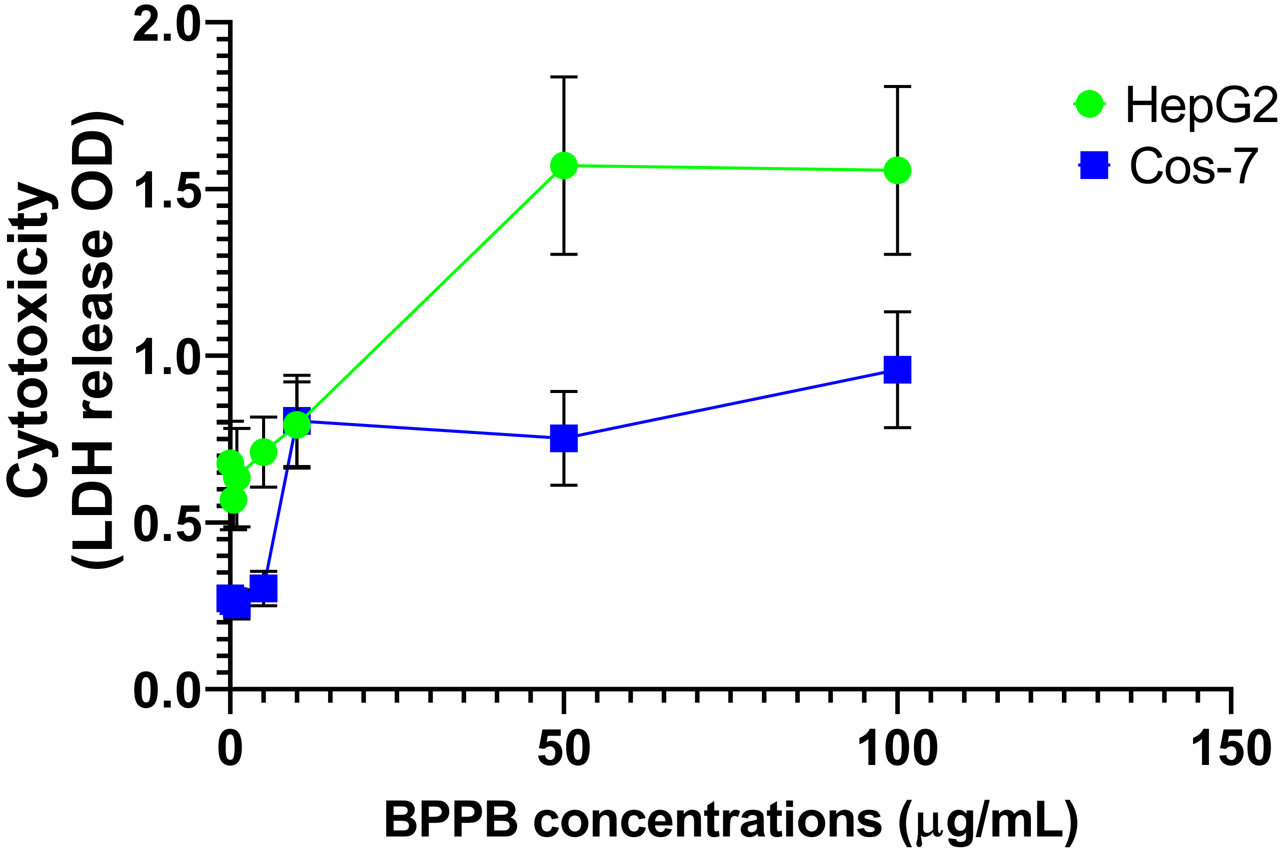

Figure S6. Cytotoxicity (LDH release OD) on Cos-7 (blue trace) and HepG2 (green trace) cells vs increasing BPPB concentrations (0.5-100 μg/mL) after 24 hours of exposure. Concentration = 0.0 μg/mL corresponded to the control.

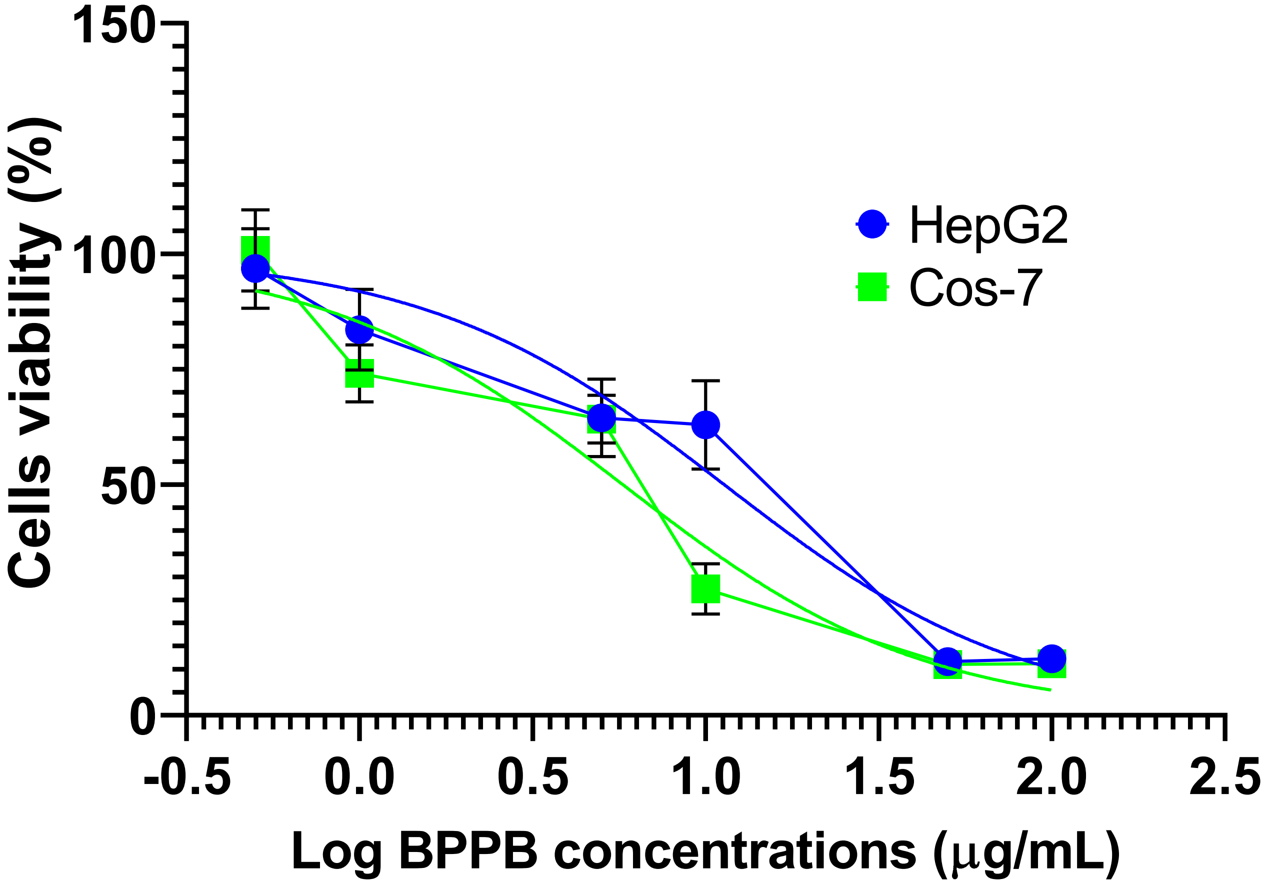

Figure S7. Plot of Log concentration of PBBP vs. cell viability (%) (blue and green traces with indicators and error bars) and plot of nonlinear fit of Log concentrations of PBBP vs. normalized response (blue and green traces without indicators).

Disclaimer/Publisher's Note: The statements, opinions and data contained in all publications are solely those of the individual author(s) and contributor(s) and not of MDPI and/or the editor(s). MDPI and/or the editor(s) disclaim responsibility for any injury to people or property resulting from any ideas, methods, instructions or products referred to in the content.
